# How good are artificial intelligence tools at identifying benign skin lesions? A systematic review and meta-analysis of the specificity of artificial intelligence tools in diagnosing suspicious skin lesions

**DOI:** 10.1093/skinhd/vzag021

**Published:** 2026-04-17

**Authors:** Suvansh Nirula, Srishti Gupta, Christian Aldridge

**Affiliations:** Faculty of Life Sciences and Education, University of South Wales, Cardiff, UK; Department of Dermatology, St George’s University Hospitals NHS Foundation Trust, London, UK; Faculty of Life Sciences and Education, University of South Wales, Cardiff, UK

## Abstract

**Background:**

Artificial intelligence (AI) is a transformative diagnostic tool in dermatology. As the prevalence of skin cancer rises and pressure on health services increases, there is an increasing demand for efficient diagnostic tools. Therefore, it is highly relevant to evaluate the diagnostic abilities of AI tools with a focus on not just the sensitivity, but also the specificity, to reduce unnecessary referrals and skin biopsies for benign skin lesions.

**Objectives:**

To evaluate the effectiveness of AI tools in diagnosing benign skin lesions, with a primary focus on calculating the sensitivity and specificity of AI tools when analysing suspicious skin lesions.

**Methods:**

A comprehensive search was conducted on multiple databases, adhering to the PRISMA 2020 guidelines. Studies with recorded sensitivity, specificity and diagnosis were included. Nine studies meeting the inclusion criteria were assessed. Data extracted included type of AI model used, sample size and important diagnostic metrics. Risk of bias was also assessed using the Quality Assessment of Diagnostic Accuracy Studies version 2 (QUADAS-2) framework.

**Results:**

Across the included studies, AI tools demonstrated a specificity of 70–95% with a pooled specificity of 82%, and a sensitivity ranging from 65% to 96% with a pooled sensitivity of 89% when analysing suspicious skin lesions as malignant vs. benign.

**Conclusions:**

AI tools possess significant potential to streamline dermatological diagnostics, especially in resource-restricted clinical setups. High sensitivity will minimize false negatives, which is crucial for early detection, and high specificity will allow the use of AI tools to autonomously discharge patients with benign skin lesions. However, there is a need for further optimization and training of AI models on more diverse datasets.

## Teaser

Artificial intelligence (AI) tools have demonstrated promise in dermatological diagnostics, especially for accurately identifying benign skin lesions. This systematic review and meta-analysis evaluate the diagnostic performance of AI models, including sensitivity and specifically focusing on specificity, highlighting their strengths and limitations across diverse clinical and teledermatology settings. High specificity (with balanced sensitivity) in AI tools can significantly reduce waiting lists and potentially allow a large number of benign skin lesions to be discharged autonomously, optimizing healthcare resource utilization.

What is already known about this topic?

- Artificial intelligence (AI) has demonstrated high sensitivity in the diagnosis of skin cancers.
- AI tools can potentially outperform primary care physicians in the accuracy of skin lesion assessment.
- The specificity of AI tools is often overlooked, but it is essential to reduce false positives and unnecessary referrals and skin biopsies.
- AI performance is highly influenced by diverse populations, the datasets used to train AI models and image quality.

What does this study add?

- We provide a systematic analysis of the sensitivity and specificity of AI tools across highly diverse clinical settings.
- We highlight various challenges that may be encountered to achieve optimal specificity in AI tools and provide guidance for future research: a high specificity would allow AI to be used as a screening tool to manage long waiting lists, with the potential to safely discharge a large number of patients with benign lesions autonomously.

The rapid development of artificial intelligence (AI) has revolutionized diagnostics in healthcare, including in dermatology.^1^ Dermatological conditions are a cause of significant burden on healthcare systems, including the UK’s National Health Service.^2^ Benign skin lesions include conditions such as melanocytic naevi, seborrhoeic keratosis, cysts and dermatofibromas.^3^ A majority of referrals made to the specialist skin cancer pathway are for benign lesions and inappropriate due to diagnostic inaccuracies.^4^ AI tools present a massive opportunity for a swift transformation of diagnostics in medicine, especially in the field of dermatology.^5^

By leveraging AI technology with high specificity and sensitivity, a significant proportion of referrals on the 2-week wait pathway could be effectively managed at the primary-care level. This would not only ease the burden on dermatology practices, but also help reduce unnecessary biopsies. AI technology also holds noteworthy promise as it can, potentially, help characterize, classify and diagnose skin lesions.^6^

AI tools, especially those using algorithms based on deep learning, for example convolutional neural networks (CNNs), have shown promising capability in the assessment of dermoscopic images of skin lesions.^7–9^ These tools have been trained to use image recognition technology to interpret images of skin lesions accurately. This training allows these models to study, identify and recognize patterns in skin lesions, and distinguish between benign and malignant skin lesions.^10^

A 2017 study conducted by Esteva *et al*. showcased that a CNN could achieve results on par with expert dermatologists in identifying skin malignancies using dermoscopic images.^11^ Another study conducted in 2018 by Haenssle *et al*. demonstrated that CNNs were able to outperform a large group of expert dermatologists in terms of sensitivity and specificity when dealing with dermoscopic images of suspected melanomas.^9^ This study concluded that physicians would benefit from using CNNs for image classification, regardless of their experience.^9^ Previously, studies analysing the diagnostic potential of AI tools using clinical and dermoscopic images have reported significantly positive results, with 61% of studies highlighting a better performance of AI in comparison with medical professionals and 29% reporting a similar performance report.^12^ Most of these studies primarily focused on accuracy and may have overlooked crucial data on specificity for the identification of benign skin lesions. High specificity is essential to minimize false positives.^13^ These false positives often result in unnecessary biopsies and increased healthcare costs, and cause excessive anxiety in patients.^14^ This systematic review aims to assess the ability of AI to diagnose benign skin lesions, and calculate the sensitivity and specificity when diagnosing suspicious skin lesions. Specificity in this scenario would be the ability of AI tools to accurately identify benign lesions; a higher number would mean a more accurate diagnosis of benign skin lesions, reducing unnecessary skin biopsies.^15^

## Materials and methods

This systematic review was carried out in adherence with the PRISMA 2020 guidelines and checklist for the reporting of systematic reviews.^16^ The study project proposal and search strategy have been registered in PROSPERO (CRD42024596074).^17^ The search strategy was outlined at the start of the review to ensure all relevant studies that meet the inclusion criteria were included in this systematic review and meta-analysis.

### Data search strategy

**Figure 1:**
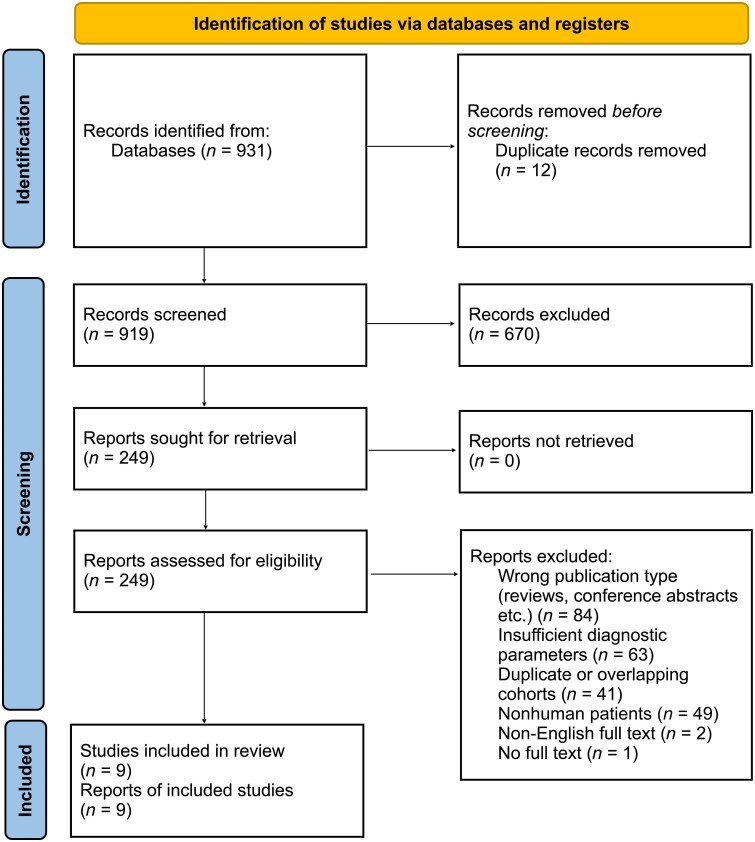
PRISMA flow diagram.^16^ This diagram shows the selection of studies included in the systematic review.

Popular databases were selected out of which two appropriate resources were finalized to be included in this systematic review: MEDLINE/PubMed and Google Scholar. PubMed was identified as the main resource for this systematic review, and advanced searches were made using Medical Subject Headings. The search for relevant articles and publications was conducted between 15 and 23 September 2024. Using a combination of PubMed and Google Scholar ensured that the search strategy was broad but relevant, in order to ensure the quality of this systematic review. Broad search terms combined using Boolean operators were able to assist with the identification of high-yield quality studies. Examples of combining terms with Boolean operators include ‘Artificial intelligence AND benign skin lesions’, ‘Artificial intelligence AND dermatology’ and ‘Artificial Intelligence AND skin cancer’. The complete search term used for this review is provided in the Supporting Information. Subsequently, search filters were applied to include articles written in English from 2014 to 2025. A PRISMA flow diagram (Figure 1) was created, which provides an outline of the study selection procedure used in this systematic review in compliance with widely accepted guidelines.^16^ To maintain a record of references, the search results were subsequently imported into EndNote20^®^.^18^ All authors independently compared study titles and abstracts obtained via databases against inclusion and exclusion criteria. The inclusion and exclusion criteria for studies included in this systematic review are provided in Table 1. The included studies evaluated two broad categories of AI tools. Firstly, clinician-facing or research-grade CNN models applied to dermoscopic or clinical images have been included. Secondly, three studies evaluated smartphone-based AI systems. All smartphone-based systems were evaluated in a supervised clinical or primary-care setup, and none of them was unsupervised use by the general population.

**Table 1:** Eligibility criteria for study selection

Inclusion criteria	Exclusion criteria
Must use AI tools like machine learning or deep learning	No use of AI tools
Studies written in English	Not written in English
Published in last 10 years	Published before 2014
Calculates diagnostic parameters like sensitivity and specificity	Does not provide key diagnostic parameters
Original articles or systematic reviews	Letter to the editor, commentary, conference abstract or conference proceedings
Only human patients	Studies with nonhuman patients

AI, artificial intelligence.

### Data extraction and synthesis

Studies that met the inclusion criteria were analysed using a structured approach and relevant data were extracted for assessment. The authors independently extracted data and assessed the risk of bias for all included studies. Extracted data included the name of database, author names, year of publication, geographical location of the study, type of study, size of the population selected, type of AI model used, method of training of AI model, type of clinical setup and relevant diagnostic parameters, including sensitivity and specificity. Other statistics like receiver operating characteristic (ROC) curve and area under curve (AUC) were also recorded. The risk of bias for each study was assessed using the Quality Assessment of Diagnostic Accuracy Studies version 2 (QUADAS-2).^19^ The elements analysed included patient selection, index test, reference standard, and flow and timing. All applicability concerns were identified for patient selection, index test and reference standard. After completion, the studies were classified into three groups: low risk, high risk or unknown risk. Data synthesis for this systematic review consisted of qualitative and quantitative analysis. A meta-analysis was conducted to summarize the overall specificity and sensitivity across all included studies. Pooling of these figures was performed using a bivariate random-effects model. Furthermore, a summary ROC (SROC) curve was generated, which aided in assessing the performance of AI tools. As a part of this model, the sensitivity and specificity are considered to be interrelated.^20^ Changes in either diagnostic parameter would likely affect the other. For example, an increase in sensitivity can result in lower specificity due to higher false positives. In the context of this study, AI tools with higher sensitivity could lead to the diagnosis of more benign lesions as malignant. In some instances, studies might show both high sensitivity and specificity, whereas some might show a single parameter as being high and the other as lower. A bivariate model was used to estimate an overall figure, including the pooled sensitivity and specificity across all studies. This approach allowed the generation of the SROC curve and area under this curve (AUROC). These plots aid in the measurement of the ability of AI tools to accurately differentiate between benign and malignant skin lesions.^21^ A qualitative or narrative analysis was conducted for studies that lacked sufficient data for quantitative synthesis or employed markedly different methodologies. This allowed including a broader range of studies, ensuring a highly comprehensive and inclusive systematic review. For example, studies using different types of AI technology, like deep learning vs. traditional machine learning, have been described narratively to emphasize on findings from each individual study.

## Results

This systematic review estimated the diagnostic accuracy of AI tools to differentiate between benign and malignant skin lesions. Nine studies comprising of a total 43 422 skin lesions were included as part of this review, as depicted in Table 2.^22–30^ Table 3 comprises data for the country where the study was conducted, the type of AI model used, the sensitivity, the specificity and the key findings for each study. A total of 17 008 lesions were malignant, whereas 26 414 lesions were benign. All studies included in this systematic review had lesions reviewed by dermatologists and AI tools, with lesions deemed to be suspicious being biopsied, and others being clinically diagnosed as benign. The review aimed to estimate the specificity of AI tools and identify key factors that may influence the diagnostic accuracy of this technology. Meta-DiSc v 2.0 (https://ciberisciii.shinyapps.io/MetaDiSc2/) was used to calculate the pooled estimates of specificity and sensitivity. A subgroup analysis and metaregression were also performed to analyse heterogeneity. Additionally, SROC curves were generated to explore heterogeneity across all studies to understand the diverse outcomes in the diagnostic performance of AI tools.

**Table 2:** Total lesions, malignant lesions and nonmalignant lesions across all studies

Variable	Value
Studies	9
Number of malignant lesions	17 008
Number of nonmalignant lesions	26 414
Total	43 422

**Table 3:** Key highlights for all included studies, with sensitivity and specificity

Study	Location	AI model	Sensitivity (%) (95% CI)	Specificity (%) (95% CI)	Key findings
MacLellan *et al.*^22^	Canada	MoleAnalyzer	0.88 (0.77, 0.95)	0.79 (0.71, 0.85)	MoleAnalyzer had better specificity than dermatologists and teledermatology services
Crawford *et al.*^23^	Canada	MoleAnalyzer	0.65 (0.38, 0.86)	0.76 (0.71, 0.80)	Comparable diagnostic accuracy for AI tools and dermatologists
Sangers *et al.*^24^	The Netherlands	SkinVision	0.87 (0.82, 0.91)	0.70 (0.66, 0.74)	High sensitivity and specificity for SkinVision. Encourages self-assessment with AI as screening tool for early cancer diagnosis
Marsden *et al.*^25^	UK	DERM (AIaMD)	0.91 (0.82, 0.97)	0.83 (0.80, 0.86)	Equivalent sensitivity and specificity for AIaMD and teledermatologists. AIaMD also showed reduced NNB
Birkenfeld *et al.*^26^	Spain	CAC system	0.84 (0.81, 0.87)	0.72 (0.69, 0.75)	Concluded that CAC AI tool can be used as a screening method for melanoma in primary health setups. ROC curve showed high potential to distinguish between benign and malignant lesions
Anderson *et al.*^27^	USA	CNN	0.80 (0.56, 0.94)	0.95 (0.88, 0.99)	CNN outperformed dermatologists. Very high specificity for CNN
Udrea *et al.*^28^	The Netherlands	SkinVision	0.96 (0.94, 0.98)	0.78 (0.77, 0.79)	Training of AI model on diverse datasets improved diagnostic accuracy
Giavina-Bianchi *et al.*^29^	Brazil	CAD system	0.90 (0.87, 0.93)	0.89 (0.87, 0.90)	Reliable diagnostic assessment by CAD systems with significant potential for use in resource-limited locations
Soenksen *et al.*^30^	USA	CNN	0.90 (0.90, 0.91)	0.90 (0.89, 0.90)	Large study with highly precise specificity evidenced by narrow CIs

AI, artificial intelligence; AIaMD, AI as a Medical Device; CAC, computer-aided classification system; CAD, computer-aided diagnosis; CI, confidence interval; CNN, convolutional neural networks; DERM, Deep Ensemble for the Recognition of Malignancy; NNB, number needed to biopsy; ROC, receiver operating characteristic.

### Study level diagnostic performance

**Figure 2:**
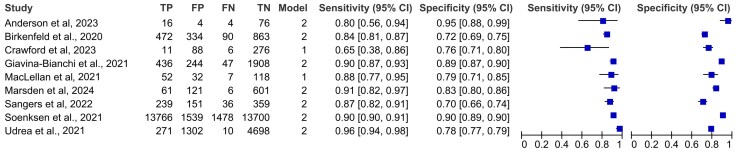
Forest plot depicting the sensitivity and specificity of artificial intelligence tools for distinguishing malignant from benign skin lesions. This figure shows study-level diagnostic accuracy figures for each included dataset. For each study, true positives (TP), false positives (FP), true negatives (TN) and false negatives (FN) are listed alongside the corresponding sensitivity and specificity values with 95% confidence intervals (CIs). The plotted squares represent point estimates, and the horizontal lines depict the 95% CI.

The sensitivity of AI tools in diagnosing malignant skin lesions across the nine included studies ranged from 65%^23^ to 96%,^28^ with studies like Soenksen *et al*.^30^ and Udrea *et al*.^28^ demonstrating high reliability (>0.9). Specificity, which measures the ability to correctly identify nonmalignant lesions, varied from 72%^26^ to 95%.^27^ Figure 2 showcases a Forest plot depicting the sensitivity and specificity across all nine studies. Narrow confidence intervals (CIs) in specificity highlight the precision of the estimated specificity, with very narrow intervals in large studies like the one conducted by Soenksen *et al*., with a total of 15 239 lesions and a specificity of 90%.^30^ Wider CIs were seen in smaller studies like that of Anderson *et al*.^27^ The variable results in terms of specificity across all nine studies may be a result of use of different AI models and algorithms, datasets used for training, skin lesion types and diverse clinical setups.

### Pooled diagnostic accuracy (meta-analysis)

Table 4 provides a comprehensive summary of the bivariate statistics for AI tools in diagnosing skin lesions. The overall sensitivity of 0.89 (95% CI 0.84–0.92) indicates a high capability of the AI tools to correctly identify malignant cases. The specificity of 0.82 (95% CI 0.76–0.97) further demonstrates their effectiveness in accurately identifying nonmalignant cases. The diagnostic odds ratio (DOR) of 36.88 (95% CI 21.02–64.68) highlights the strong association between AI-generated diagnosis and the true disease status of the patient. This demonstrates the ability of AI technology to correctly differentiate malignant from benign lesions, although it should be noted that the DOR is distinct from overall accuracy. The positive likelihood ratio of 5.02 (95% CI 3.69–6.82) confirms the effectiveness of AI tools in verifying positive cases, while the negative likelihood ratio of 0.14 (95% CI 0.09–0.19) underscores their proficiency in ruling out negative cases. Additionally, the false positive ratio of 0.18 (95% CI 0.13–0.23) indicates a relatively low incidence of nonmalignant cases being misclassified as malignant.

**Table 4:** Bivariate summary statistics

	Value	95% confidence interval
Sensitivity	0.89	0.84–0.92
Specificity	0.82	0.76–0.87
Diagnostic odds ratio	36.88	21.02–64.68
Positive likelihood ratio	5.02	3.69–6.82
Negative likelihood ratio	0.14	0.09–0.19
False positive rate	0.18	0.13–0.23

### Summary receiver operating characteristic curve analysis

**Figure 3:**
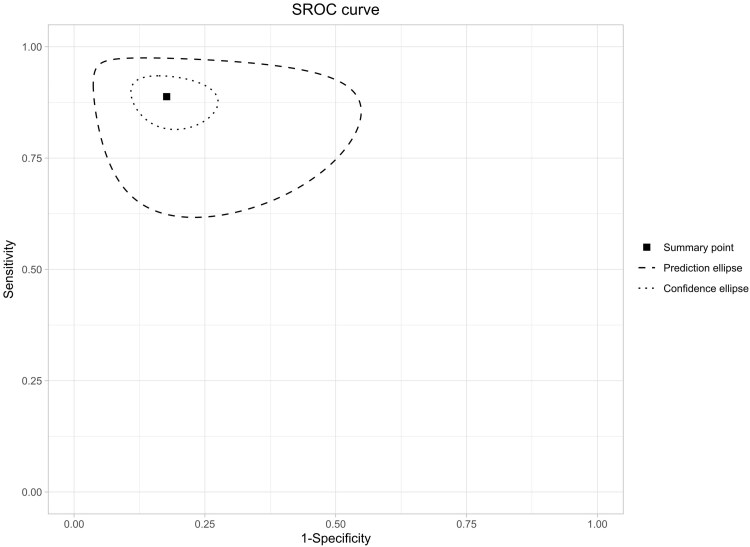
Summary receiver operating characteristic (SROC) curve for the diagnostic performance of artificial intelligence (AI) tools. The SROC plot depicts the pooled sensitivity on the vertical axis and 1 – specificity on the horizontal axis, which have been calculated from the bivariate meta-analysis of all included studies. The central marker highlights the combined diagnostic accuracy of the AI models in distinguishing malignant from benign skin lesions (overall summary point). The dashed ellipse demonstrates the prediction region, indicating the expected set of results for a new study. The dotted ellipse showcases the 95% confidence region around the summary point. A curve approaching the upper-left corner indicates high sensitivity and specificity across studies.

An SROC curve was generated (Figure 3) that visualizes diagnostic performance by plotting sensitivity against the false positive rate (1 – specificity). An ideal diagnostic method would approach the top-left corner, indicating high sensitivity and specificity. The curve created below the performance by AI tools is approaching the top-left corner, which suggests high accuracy when analysing skin lesions. The AUC demonstrates higher values, suggesting good performance. This systematic review highlights variability across AI tools; for example, Udrea *et al*. reported high sensitivity for true positives,^28^ while Soenksen *et al*. showed high specificity for true negatives.^30^ Large studies like that by Soenksen *et al*. had narrow CIs, which suggests precise estimation of specificity,^30^ whereas studies reporting lower specificity like that of Birkenfeld *et al*. highlight methodological variations.^26^

### Risk of bias and applicability

As shown in Table 5, the risk of bias (QUADAS-2 score) and applicability concerns for each study were assessed. Overall, the risk of bias across the included studies is low. Some concerns regarding applicability have been discussed, especially the lack of diverse skin types.

**Table 5:** Risk of bias and applicability concerns for each study

Study	Risk of bias (QUADAS-2)	Applicability concerns
MacLellan *et al.*^22^	Low	Low
Crawford *et al.*^23^	Moderate (selection bias as self-referred patients)	High: variability in image quality and exclusion of lesions in inaccessible areas
Sangers *et al.*^24^	Low	High: lack of diverse skin types and exclusion of poor-quality images underestimates real world challenges
Marsden *et al.*^25^	Low	Low
Birkenfeld *et al.*^26^	Low	Low
Anderson *et al.*^27^	Low	Low
Udrea *et al.*^28^	Low	High: lack of diverse skin types and exclusion of poor-quality images underestimates real world challenges
Giavina-Bianchi *et al.*^29^	Low	Low
Soenksen *et al.*^30^	Low	Low

QUADAS-2, Quality Assessment of Diagnostic Accuracy Studies version 2.

## Discussion

This systematic review showcases the potential of AI tools in distinguishing benign from malignant skin lesions. The pooled sensitivity and specificity results are comparable to results from other meta-analyses and systematic reviews. For instance, the pooled sensitivity of 89% and specificity of 82% from this systematic review is in line with the outcomes from Krakowski *et al*., who showed that clinicians achieved a pooled sensitivity of 81.1% and specificity of 86.1% with AI assistance, whereas for clinicians without AI assistance, the pooled sensitivity was 74.8% and specificity was 81.5%.^31^ Krakowski *et al*. concluded that AI improved the performance of all clinicians, with nondermatologists benefiting the most from AI assistance.^31^ Additionally, Brinker *et al*. reported that AI tools can perform at the same level or outperform board-certified dermatologists when being evaluated under controlled clinical conditions.^32^ Esteva *et al*. reported a performance similar to dermatologists, with CNNs achieving an AUC of 0.96 when classifying skin lesions.^11^ Salinas *et al*. conducted a meta-analysis of 19 studies and calculated that AI can diagnose skin cancer with a sensitivity of 87% and specificity of 77%.^12^ These comparisons reiterate that the results of this systematic review are robust and highlight that augmentation of clinical diagnosis through AI support is evolving into a reliable tool in dermatological practice.

The findings of this review also showcase the real-world relevance beyond diagnostic parameters. By ensuring high sensitivity and specificity, AI tools can assist in triaging skin lesions by highlighting suspicious lesions that may be malignant while helping clinicians to safely discharge lesions that are benign, reduce pressures on dermatology departments and avoid unnecessary skin biopsies.

It is vital to clarify the types of AI tools represented in this systematic review. The majority of studies in this review evaluated clinician-facing or research-grade AI models applied to dermoscopic images. Sangers *et al*., Udrea *et al*. and Giavina-Bianchi *et al.* assessed smartphone-based systems, although all were evaluated within clinical or primary-care setups rather than unsupervised, at-home patient use.^24,28,29^

In addition to supervised clinical evaluation, AI technology is being developed to allow broader access, including tools being made for direct-to-consumer use. Smartphone applications (‘apps’) help patients to take images of their skin lesions and use AI technology to provide a quick risk assessment. Börve *et al*. showcased that patients could easily capture and transfer dermatological pictures using their phones.^33^ However, there are safety concerns when these apps are used without supervision. Sun *et al*. evaluated 25 commercially available apps and concluded that the mean sensitivity was 28%, mean specificity was 81%, eight apps were unsuccessful in identifying a single melanoma in their top-1 differential diagnosis ranking and four apps did not place melanoma in their top-3 ranking.^34^ Wolf *et al*. analysed the performance of four smartphone apps and concluded that three of them wrongly classified 30% of patients with melanomas as having benign skin lesions and, therefore, unsupervised use could delay the diagnosis of or misdiagnose skin malignancies, and should not replace clinician assessment.^35^ Freeman *et al*. conducted a systematic review that evaluated the diagnostic accuracy of algorithm-based smartphone apps and found significant limitations in real-world reliability.^36^ Across the studies included, most apps showed high variability in diagnostic performance in the detection of skin cancer. Freeman *et al*. also concluded that the performance reduction is expected when the apps are used by end-users and in clinically relevant populations.^36^

The performance of AI tools must be interpreted cautiously. Wide CIs in studies with smaller samples demonstrate issues with generalized application in clinical settings. Nagendran *et al*. also highlight that there are limited datasets and source code available, which can make it difficult to interpret, verify and reproduce results from research pertaining to the use of AI tools in dermatological diagnosis.^37^ Therefore, there is a need for extensive validation and research in demographically diverse patient samples and clinical scenarios before AI tools are routinely used in primary- and secondary-care setups.

To summarize, this systematic review concludes that AI tools present significant promise for triaging and diagnosing skin lesions, especially in resource-limited primary-care setups. Research in the future should focus on analysing skin lesions on a larger scale across highly diverse patient populations to ensure efficient and medically safe use of AI in real-world clinical practice.

This systematic review followed the PRISMA guidance and the studies included followed dermatologist-confirmed reference standards, therefore increasing reliability of the pooled sensitivity and specificity. A key strength of this review is the focus on studies using dermatologist-verified diagnosis, ensuring that the performance of AI tools was compared against robust clinical benchmarks. The review also provides an up-to-date analysis of diagnostic accuracy across various AI systems. A limitation of the review is that the evidence base remains small, and most studies involve specialist dermatology settings, which can limit application to broader clinical setups.

### Author contributions

Suvansh Nirula (Conceptualization [lead], Data curation [lead], Formal analysis [lead], Investigation [lead], Methodology [lead], Project administration [lead], Software [lead], Validation [lead], Writing—original draft [lead], Writing—review & editing [lead]), Srishti Gupta (Conceptualization [supporting], Data curation [supporting], Formal analysis [equal], Investigation [equal], Methodology [equal], Project administration [equal], Writing—original draft [equal], Writing—review & editing [equal]), and Christian Aldridge (Conceptualization [lead], Data curation [supporting], Formal analysis [supporting], Funding acquisition [supporting], Investigation [supporting], Methodology [equal], Project administration [lead], Resources [lead], Supervision [lead], Writing—original draft [supporting], Writing—review & editing [lead])

## Supplementary Material

vzag021_Supplementary_Data
