# Supplementary material for: How good are artificial intelligence tools at identifying benign skin lesions? A systematic review and meta-analysis of the specificity of artificial intelligence tools in diagnosing suspicious skin lesions
Source: Skin Health Dis. 2026 Apr 17;6(3):224–31. doi: 10.1093/skinhd/vzag021 (PMC13220024; doi:10.1093/skinhd/vzag021)
Supplement: vzag021_Supplementary_Data [file vzag021_supplementary_data.docx]

Search Criteria

The search strategy was developed by the authors of this systematic review. Broad search terms combining artificial intelligence related terms with dermatology and skin cancer terminologies were used. Boolean operators were applied to identify highly relevant and high-yield publications.

The main search strings included combinations of:

- AI related terms: “artificial intelligence,” “machine learning,” “deep learning,” “neural networks,”
- Skin cancer terms: “skin cancer,” “melanoma,” “skin lesion,” “benign skin lesions,” “basal cell carcinoma,” and “squamous cell carcinoma.”

One of the main search string used on Pubmed:

("artificial intelligence" OR "machine learning" OR "deep learning" OR "neural network*" OR "convolutional neural network*" OR "CNN" OR "computer vision" OR "automated diagnosis" OR "algorithm*" OR "diagnosis, computer-assisted" OR "pattern recognition, automated") AND ("skin neoplasm*" OR "melanoma" OR "skin cancer" OR "basal cell carcinoma" OR "squamous cell carcinoma" OR "non-melanoma skin cancer" OR "pigmented lesion*" OR "skin lesion*" OR "nevus" OR "naevi" OR "dysplastic nevus" OR "actinic keratosis") AND ("diagnosis" OR "sensitivity" OR "specificity" OR "accuracy" OR "predictive value of tests" OR "ROC curve" OR "area under curve" OR "AUC" OR "diagnostic performance" OR "validation study") Filters: Abstract, Free full text, Full text, Adaptive Clinical Trial, Case Reports, Classical Article, Clinical Study, Clinical Trial, Clinical Trial Protocol, Controlled Clinical Trial, Editorial, Guideline, Meta-Analysis, Multicenter Study, Network Meta-Analysis, Observational Study, Pragmatic Clinical Trial, Randomized Controlled Trial, Review, Scoping Review, Systematic Review, Validation Study, English, Humans, from 2014 – 2024

One of the main search string used on Google Scholar:

("artificial intelligence" OR "machine learning" OR "deep learning" OR "convolutional neural network" OR "neural network" OR "computer vision" OR "automated diagnosis" OR "algorithm") AND ("skin cancer" OR "melanoma" OR "basal cell carcinoma" OR "squamous cell carcinoma" OR "pigmented lesion" OR "skin lesion" OR "nevus" OR "naevi") AND ("diagnosis" OR "sensitivity" OR "specificity" OR "accuracy" OR "ROC curve" OR "area under curve")

Further filters were applied to narrow down the search as depicted in the PRISMA flow diagram
